# The qualitative analysis of characteristic of callers to a psychological hotline at the early stage of COVID-19 in China

**DOI:** 10.1186/s12889-021-10883-w

**Published:** 2021-04-28

**Authors:** Na Du, Yingjie Ouyang, Zongling He, Juan Huang, Die Zhou, Yin Yuan, Yunge Li, Manxi He, Yong Chen, Hongming Wang, Yuchuan Yue, Maoxiang Xiong, Keliang Pan

**Affiliations:** 1 Department of clinical psychology, The Fourth People’s Hospital of Chengdu, Huli west 1 alley NO.8, Jinniu district, Sichuan province 610039 Chengdu, China; 2 Department of medical, The Fourth People’s Hospital of Chengdu, Sichuan province 610039 Chengdu city, China

**Keywords:** Mental state, Psychological hotline, Public, COVID-19

## Abstract

**Background:**

As the outbreak of COVID-19, traditional face-to-face psychological intervention are difficult to achieve, so hotline becomes available and recommended strategies. The callers’ characteristic could help us to study their experiences of emotional distress, as well as the reasons for calling during the pandemic, which can be used to inform future service design and delivery.

**Methods:**

The information of 1558 callers called our hospital’ s hotline for help from February 3, 2020, to March 16, 2020 were collected in the form of Tick-box and Free text, and the inductive content analysis was undertaken focusing on the reasons for caller engagement.

**Results:**

It was indicated that more than half of the callers are female (59.7%), mostly between the age of 18–59 (76.5%). The average age was 37.13 ± 13.76 years old. The average duration of a call to the hotline was 10.03 ± 9.84 min. The most frequent description emotional state were anxious (45.1%) and calm (30.3%), with the sub-sequence of scared (18.2%), sad (11.9%), and angry (6.9%). All callers displayed a wide range of reasons for calling, with needing support around their emotion (64.6%), seeking practical help (44.0%), and sleep problems (20.3%) constituting the majority of calls. Among the subthemes, 314 callers thought the epidemic has made them upset, 198 asked questions about the epidemic, and 119 reported their life routines were disrupted. The prevalence of key reasons does not appear to differ over time. Through their feedback, 79.1% agreed that they felt emotionally better after calling, and 95.0% agreed that hotline had helped them.

**Conclusions:**

During the epidemic, the most concern of the public is still related to epidemics and its adverse effects. Fortunately, the hotline can be an active and effective rescue measure after an emergency happened.

## Background

The COVID-19 epidemic that was firstly found in Wuhan has now spread to all parts of the world and has not yet been eliminated. This is an urgent public health emergency that poses a threat to human health and life safety, including severe economic, social, and psychological shocks. This is also a severe challenge for the public health system of our city. It damages the health of thousands of people and has a serious psychological impact on the public [1]. This highly contagious virus causes anxiety, panic, and other negative emotions to spread and accumulate in society [2]. All kinds of information about the epidemic situation from various sources might also aggravated the inner fear of the public and harmed their mental health [3]. Therefore, public mental health assessment and counseling services are undoubtedly essential in such public health emergencies [4].

Due to the particularity of the COVID-19 pandemic, traditional face-to-face psychological interventions and consultations are challenging to achieve, limiting the assessment and intervention methods. Ommeren et al. suggested that most acute stress problems in emergencies are best treated without medication following the principles of psychological first aid [5]. In this case, if online technologies such as assistance hotline and online counseling can be fully utilized, specialists can quickly assess the mental health level of the clients and provide corresponding psychological intervention services. Research has revealed that the effectiveness of telephone-delivered psychotherapy was similar to traditional face-to-face therapy [6], and previous research reported that telephone hotline could provide specialized mental health support to families [7]. It also has the potential to overcome barriers, including geographical isolation [8], fear of stigmatization, and difficulty accessing transportation [9]. Facts have proved that the hotline can reduce the pressure of consultation and provide a safe environment for people to express their emotions [10]. Due to its multiple advantages, the hotline has become one of the most popular crisis intervention measures globally [11]. Therefore, considering the particular circumstances of the epidemic, the assistance hotline may become the best and most convenient way of rescue.

Under such circumstances, various forms of mental health services conducted on line have emerged in China [12]. However, the practice in China indicates that the importance of hotline cannot be replaced by the newly developed online services [13]. There is concrete evidence to prove the effectiveness of the assistance hotline. Mishra and Daigle pointed out that 14% of the callers had their depressive mood reduced after the hotline call [14]. In addition, during the hotline call, it was observed that the caller’s mental state (including confusion, anger, anxiety, and helplessness) significantly improved [15, 16]. Generally speaking, hotline can effectively provide a platform for the public to deal with their psychological stresses, and can supply them with instrumental and informational help [13]. More importantly, it may ease the pressures on the city’s overburdened emergency medical services during the epidemic [17].

At the beginning of the epidemic, as the mental health center of the city, the Fourth People’s Hospital of Chengdu first assumed the responsibility of protecting the mental health of the public. It immediately expanded the number of original psychological assistance hotline and extended its working hours. Although our hospital’s hotline has been serving the public for a long time, the past data of them has not been carefully analyzed, including the demographic information of the callers and the reasons for their calls. The sharp increase in the number of calls during the epidemic reflects the significant increase in public demand for aid during special periods. Therefore, we adopted a retrospective and descriptive method to analyze the data related to these calls during the COVID-19 outbreak. The purpose is to explore the psychological state of people in the epidemic, as well as the contribution and situational factors that lead to their calling. In this way, we can indirectly understand the issues they are more concerned about during the outbreak and how they will affect their lives. The caller’s feedback can also provide evidence of whether the hotline is effective during the epidemic.

## Methods

### The design of the study

Because of the characteristics of the hotline, we could not obtain the objective scores on their emotions assessed by corresponding scales. To ensure the accuracy of their emotional state after the pandemic, we made the decision of using the method of intake form to record their self-deemed emotions and their reasons for calling. Although the hotline was set up to relieve the pressure of the public, and we would like to know the actual effect of the hotline, we have added the feedback part to our intake form. The data came from the oral description of the callers, so the inductive content analysis was the main method to further analyze their calling reasons.

### The operation of the hotline

The contact information of the hotline was disseminated to the public through the Internet, media, and radio stations to help people deal with their psychological problems during the outbreak. A professional team of 15 people operates the assistance hotline, 8 are psychologists, and the rest are clinical psychologists with psychotherapist qualifications. The above-mentioned personnel all have a bachelor’s degree or above and have practical psychological counseling experiences. Before answering the hotline, they received unified hotline service training. The hotline was divided into three landlines, which were answered in different rooms. The hotline provides telephone consultation and crisis intervention services with toll-free lines operating 24 h a day, 7 days a week. For every call received, the operator needs to fill out a call intake form to record demographics information and essential descriptive characteristics of the calls. Operators were trained on the usage of these intake forms to increase their reliability. These intake forms were then entered into an online database specifically designed to store this data.

### The context of the hotline intake form

Our intake form was completed in the form of “Tick-box” and “Free-text.” Tick-box data includes gender, caller’s mental state (e.g., anxious, sad, calm), psychiatric history, and risk of harm to themselves and others. Those callers at risk would be referred to further video psychological crisis intervention treatment or be proposed to the hospital for treatment. If they were committing suicide, the operator would contact their family members and the police to find them to avoid death. The purpose of the free-text was to collect the specific reasons for their callings, and the information of age, occupation, date of the call, and call duration were also recorded in it. Although the free-text data are recorded by the operator, the organizational policy is that the content of these records is based on the caller’s conversation, which is to ensure that it reflects the original content of the caller’s statement. At the end of each call, all callers were asked to give their feedback on the hotline’s effectiveness, including their opinions on the hotline and how they feel after contacting the hotline.

### Data collation and analysis

On January 30, 2020, the World Health Organization declared the outbreak of COVID-19 a public health emergency of international concern [18]. After realizing the seriousness of the situation, our hospital opened the hotline within only 3 days of preparation. Therefore, we collected data from February 3, 2020, to March 16, 2020. During this period, the pandemic was most prevalent in China, with a total number of infections of 80,881 (https://baike.baidu.com/item/2020%E5%B9%B4%E4%B8%AD%E5%9B%BD%E6%96%B0%E5%86%A0%E8%82%BA%E7%82%8E%E7%96%AB%E6%83%85%E5%8F%91%E5%B1%95%E5%AE%9E%E5%BD%95/50157160?fromtitle=2020%E5%B9%B4%E6%96%B0%E5%86%A0%E8%82%BA%E7%82%8E%E7%96%AB%E6%83%85%E5%8F%91%E5%B1%95%E5%AE%9E%E5%BD%95&fromid=24334213#reference-[83]-25605585-wrap). Since then, most companies have declared to resume work, and the number of calls was relatively low, with an average of 5 per day. Thus, we did not use subsequent data for analysis. For telephone calls, we can only consider it to be effective only after filling out the intake form. The Ethics Committee of the Fourth People’s Hospital of Chengdu approved this study.

For quantitative data, the analysis involves descriptive statistics, with data expressed in frequency and percentage. One of the qualitative evaluations, the inductive content analysis, was utilized to identify the main themes across the data set. They read the recorded free text of each caller independently and wrote as many headings as necessary to describe all possible reasons. After this, the lists of headings are grouped under higher order subthemes. At first, there were 32 subthemes, and to meet the tenets of creditability and trustworthiness, these subthemes were discussed collectively by the 3 members mentioned above until consensus was reached. As a result, similar subthemes were merged to prevent overlap of categories, and 21 subthemes were retained. Then the 4 key overarching themes were abstracted from the 21 subthemes using content-characteristic words.

## Results

### Total number of calls per day

**Fig. 1:**
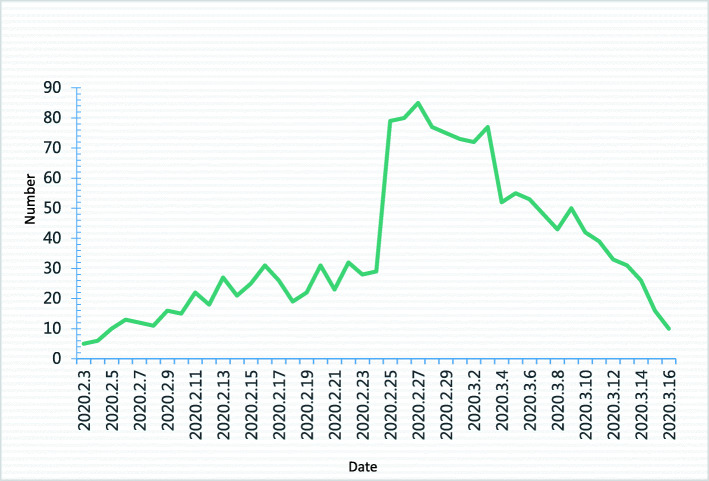
Total number of calls per day

Figure 1 summarizes the daily call distribution, showing that the number of calls per day varies greatly, from a minimum of 5 calls per day to a maximum of 85 calls, with a mean of 37.10 calls per day. The average duration of a call is 10.03 ± 9.84 min (minimum = 1; maximum = 108 min).

### Caller characteristics

Of the 1558 intake forms we recorded, 40.3% of callers were male (628/1558), and 59.7% were female (930/1558). Among them, 230 people were unwilling to reveal their age. Moreover, of the remaining 1328 people, the oldest was 89 years old, the youngest was 12 years old, and the average age was 37.13 ± 13.76 years old. According to WHO’s age classification (https://www.360kuai.com/pc/9cb0ccfb0ac2ca07f?cota=4&kuai_so=1&tj_url=so_rec&sign=360_57c3bbd1&refer_scene=so_1), there were 40 callers at the age of 12–17 years old (teenager), accounting for 2.6%; 934 callers at the age of 18–44 years old (youth), accounting for 59.9%; 259 callers at the age of 45–59 years old (middle-aged), accounting for 16.6%; and 95 callers at the age of 60 years old or above (elderly), accounting for 6.1%.

When recording the caller’s perceived emotional state during the call, the operator used the tick-box section of the intake form. Simultaneously, the operator needed to collect the corresponding information that could reflect caller disclosure, and made some assessment based on the caller’s self-report. Among all eligible intake forms, the most frequently mentioned emotions are anxious (45.1%) and calm (30.3%) (see Table 1 for details). Further analysis suggested that 158 callers (10.1%) had two kinds of emotions, 14 (0.90%) had three kinds of emotions and 2 (0.13%) with four different emotions.

**Table 1:** Caller characteristics, statement of emotion, psychiatric history and identified risk at intake

	Total (***n*** = 1558)
	n (%)
**Gender**	
Male	628 (40.3)
Female	930 (59.7)
**Age** ^**b**^	
12–17	40 (2.6)
18–44	934 (59.9)
45–59	259 (16.6)
> 60	95 (6.1)
**Emotion** ^**a**^	
Anxious	702 (45.1)
Scared	283 (18.2)
Sad	186 (11.9)
Angry	108 (6.9)
Calm	472 (30.3)
**Psychiatric history**	
Anxiety disorder	32 (2.1)
Depression	23 (1.5)
Bipolar disorder	14 (0.9)
Obsessive compulsive disorder	9 (0.6)
Post-traumatic stress	4 (0.3)
Other mental health history (schizophrenia,personality disorder … …)	16 (1.0)
Some mental health history, non-specific	13 (0.8)
**Identified risk**	
Self-harm	5 (0.3)
Suicidal thoughts	3 (0.2)
Harm to others	1 (0.0)
Alcohol and/or other drugs	6 (0.4)

Note. ^a^ Multiple options could be selected, therefore percentages may add to greater than 100

^b^ There were callers unwilling to reveal their age, therefore percentages may add to lower than 100

### Psychiatric history and risk assessment

One hundred eleven callers (7.1%) reported a history of mental illness, the most common of which were anxiety, depression, and bipolar disorder (Table 1). It is unknown whether callers who have reported a history of a psychiatric disease have previously received a formal diagnosis.

When a caller experiences a severe decline in mental health, which affects his or others’ health and life safety, the operator will record an assessment of “at-risk” based on the caller’s self-reporting. After assessment, 15 callers (0.96%) were found to be impacted by risks. The distribution and the risk form are shown in Table 1. Most commonly, callers were assessed as being at risk due to their inappropriate behaviors, including alcohol and/or other drugs (40%) and self-harm (33.3%).

### Reasons for calling the hotline

After analyzing the free-text section about the callers’ reasons for calling, 21 subthemes were identified, and four key overarching themes were abstracted at last, including seeking practical help, the need for support around their emotion, sleep problems, and consulting with others’ issues instead of themselves. When looking at the prevalence of themes, most calls sought support around their emotion (*n* = 1007, 64.6%). The second most common theme of calls was seeking practical help (*n* = 685, 44.0%) (See Table 2 for details). To make the subthemes more intuitionistic and specific, we extracted representative statements from the intake forms for the 21 subthemes, respectively. (See Table 3 for details).

**Table 2:** Frequency of themes on the reasons for calling the hotline

Themes	Subthemes	Frequency n (%)^a^
**Seeking practical help**	Ask questions about the epidemic itself	198 (12.7)
	Ask how to protect themselves from being infected	156 (10.0)
	Ask whether their symptoms stood for infection	101 (6.5)
	Ask whether they had developed mental disorders	87 (5.6)
	Ask information about hospitals and medical care	74 (4.7)
	Ask the new policy under the epidemic	45 (2.9)
	How to educate their kids	24 (1.5)
	**Total Seeking practical help**	685 (44.0)
**Needing support around their emotion**	The epidemic has made them upset	314 (20.2)
	Uncomfortable when knowing the latest information of COVID-19	197 (12.6)
	Worried about their life condition including work, study	134 (8.6)
	Worried about their family members’ health	96 (6.2)
	Scared of being isolated	67 (4.3)
	Angry about someone without protective measures	65 (4.2)
	Worried about the recurrence of their psychiatric disease	47 (3.0)
	Angry about the shortage of protective material	43 (2.8)
	Problems of interpersonal relationship, including friends, lovers, family members, colleagues	35 (2.2)
	Missing of their dead loved ones	9 (0.6)
	**Total Needing support around their emotion**	1007 (64.6)
**Sleep problems**	Life routines were disrupted	119 (7.6)
	Changed emotion made them hard to sleep	105 (6.7)
	Specific events made them hard to sleep	67 (4.3)
	Relapse of pre-existing mental illness	26 (1.7)
	**Total Sleep problems**	317 (20.3)
**Consulting with others’ issues instead of themselves**	**–**	11 (0.7)

^a^ Multiple options could be selected, therefore percentages may add to greater than 100

**Table 3:** The representative statements from the intake forms for the 21 subthemes

Subthemes	Representative statements
1. Ask questions about the epidemic itself	1. “When will the epidemic end?”
2. Ask how to protect themselves from being infected	2. “How to wear the mask correctly, and is it safe to buy food in the supermarket?”
3. Ask whether their symptoms stood for infection	3. “I have a sore throat and headache, and does it mean I am infected with COVID-19?”
4. Ask whether they had developed mental disorders	4. “I feel out of control, and I am confused by my involuntary worries. Have I suffered from depression?”
5. Ask information about hospitals and medical care	5. “Is your hospital still open for business? I want to prescribe some medicine.”
6. Ask the new policy under the epidemic	6. “Is the highway still open during the epidemic?”
7. How to educate their kids	7. “My kids are not obedient at all. How to teach them to become well-behaved?”
8. The epidemic has made them upset	8. “I feel so upset because of the epidemic, and I can’t control myself worrying about it.”
9. Uncomfortable when knowing the latest information of COVID-19	9. “Every time I seeing the death toll caused by the epidemic, I feel so sad.”
10. Worried about their life condition including work, study	10. “I have to stop my work because of the epidemic, and no work means no salary. How could I survive?”
11. Worried about their family members’ health	11. “My son is living in Hubei now, and I am so worried about his health. If he is infected, what should I do?”
12. Scared of being isolated	12. “If I get infected, I have to be quarantined. That is too scary.”
13. Angry about someone without protective measures	13. “Why someone on the street does not wear masks? It’s immoral behaviour. I am so angry seeing this.”
14. Worried about the recurrence of their psychiatric disease	14. “I was diagnosed with an anxiety disorder before, and I feel nervous recently. Has my disease recurred?”
15. Angry about the shortage of protective material	15. “The shortage of masks and gloves made me very angry. Without these materials, how could I protect myself?”
16. Problems of interpersonal relationship, including friends, lovers, family members, colleagues	16. “My girlfriend and I are on the rocks. What should I do to recover our relationship?”
17. Missing of their dead loved ones	17. “My father passed away two weeks ago. I miss him so much, but there is nothing I could do.”
18. Life routines were disrupted	18. “I do not need to get up early because of the epidemic. I sleep upside down.”
19. Changed emotion made them hard to sleep	19. “The anxiety about the epidemic made it difficult for me to sleep.”
20. Specific events made them hard to sleep	20. “I could not go abroad to talk about the contract. When thinking about this issue, it’s hard for me to fall asleep.”
21. Relapse of pre-existing mental illness	21. “My depression has recurred, and I could not fall asleep again.”

**Fig. 2:**
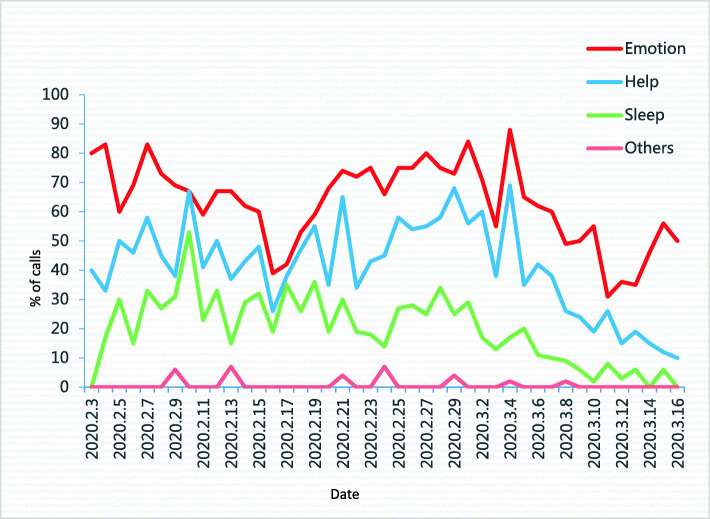
Percentage prevalence of themes over time

In Fig. 2, we can tell that the prevalence of key themes does not show obvious difference over time. Although the peaks and troughs of the frequency can be seen, calls related to needing support around their emotion are consistently more prevalent than other themes.

### Feedback on the hotline

**Fig. 3:**
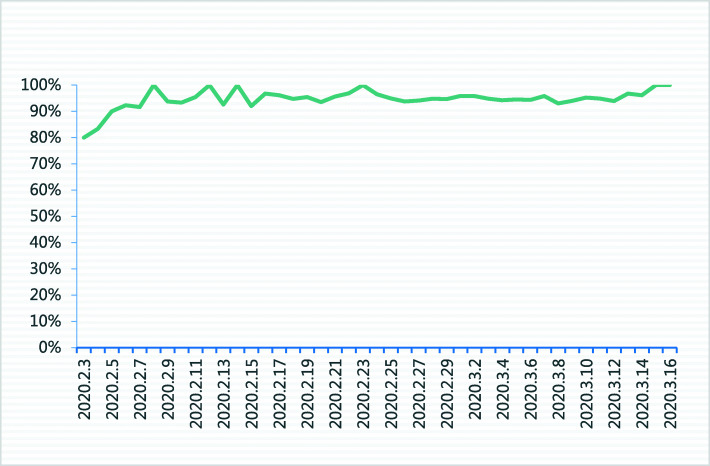
Percentage of people who thought the hotline was helpful over time

98.1% (1529/1558) of the respondents “agree” that the operator who answered the hotline understood their concerns, while 79.1% (797/1007) of the respondents said that they would feel emotionally better after talking to the operator. A total of 95.0% (1480/1558) agreed that the hotline helped them overall. When exploring the changing tendency of the feedback on the hotline, we calculated the percentage of people who thought the hotline was helpful for each day. We found that the effect of the hotline progressed smoothly, with the average effective rate of 94.7%. (see Fig. 3 for details)

## Discussion

The COVID-19 is a sudden pandemic with powerful infectivity and unpredictability. It is a strong stressor for both individuals and society. Although Sichuan Province was not a severely affected area, the government had also adopted countermeasures such as first-level public health response and lockdown to prevent and control the epidemic, which brought inconvenience to daily life and outdoor activities. Therefore, it is inevitable that some people will encounter apparent mental troubles. On the one hand, the establishment of the psychological assistance hotline in this situation can study and track the mental status of the public and obtain information about their concerns during the epidemic. On the other hand, it can provide psychological services to the people in time to prevent their psychological problems from further deteriorating and appropriately alleviate their panic.

According to the hotline data chart, we can see that the hotline can be divided into two stages according to the number. Although we have opened the hotline on February 3, 2020, and promoted it through our online official social account and broadcasting, the number of calls in the early stage was still small. To reach more people, we expanded our publicity since February 25, 2020, by sending text messages to all mobile phone users in our city, after when the number of hotline reached the peak. This means that when encountering the same type of emergency, it is crucial to consider how people obtain information. Even in the Internet age, some people do not even know how to use smartphones, and they cannot get help in time. We can use every means that we can think of to promote the hotline so as to make it available to every citizen.

First of all, in terms of the impact of the epidemic on seeking the help of different genders, the ratio of males to females in the 1558 effective calls received was 1:1.48, which is similar to Gao et al.’s statistics of the counseling hotline established by the Chinese Academy of Sciences during SARS [19], whose results showed that more women than men were seeking for assistance during an epidemic. A possible explanation for this phenomenon may be related to the richer emotional experience of women, who are more willing to express their emotions after undergoing stressful events [20]. However, due to the lack of emotional expression of men, this may lead to incorrect assessment and identification of their psychological problems. From this perspective, it is necessary to pay more attention to their corresponding mental and behavioral responses.

An interesting finding is that the age distribution of callers is concentrated in the youth and middle age. On the one hand, for other groups, teenagers may focus more on their studies and pay less attention to major social events. While the elderly are not good at obtaining valuable and helpful information from the media or social networks [21]. Therefore, the proportion of incoming calls corresponding to these groups seems to be very low. Generally speaking, the elderly are more prone to physical, mental, or senile diseases. However, due to the outbreak of this epidemic, large-scale isolation and restrictions on public transportation inevitably reduced their opportunities for diagnosis and treatment [22]. This also poses a massive challenge to the mental health services of the elderly in the community. During the crisis, the psychological services established in China in the past seemed to pay insufficient attention to the elderly group [22]. Due to modern technology limitations for the elderly, teleconsultation should be fully utilized, which is an effective way to supply the elderly with health services.

We also found that in the context of the epidemic, the public showed obvious anxious emotion. This implies that the epidemic did have a certain negative impact on public emotional changes. Similar to the SARS outbreak in 2003, the COVID-19 outbreak is considered a strong stressor. This large-impact stressor directly caused some individuals and groups to suffer from various degrees of psychological crisis [23]. A parallel research during the SARS epidemic discovered that 13.9% of the people from different groups expressed mild anxiety about this event [24]. Furthermore, according to the investigation by Ding et al., it was found that there was indeed a public psychological crisis during the SARS epidemic, and it was more serious [25]. We found that among people with emotional distress, the second most common emotion is fear. After the outbreak, the rising number of confirmed cases, news reports, and various unrecognizable social media information continued to increase, which will undoubtedly intensify the social panic [26]. Due to the shortage of medical supplies for households and daily necessities, supermarkets, pharmacies, and other stores were quickly sold out, which further exacerbated people’s panic [27]. Just like Zhang’s report, they found that more than half of the participants in their survey expressed fear due to the COVID-19 pandemic [28].

Besides, the study discovered that some people are emotionally complicated when they call the hotline. In some cases, anxiety, fear, sadness, and anger can even occur simultaneously. As Garcia et al. discovered that under tremendous pressure, people would feel consciously unwell, accompanied by low mood, alienation, and hostility [29]. Bai et al. also suggested that in a major public health emergency, the symptoms of somatization, depression, terror, and paranoia increase significantly in public [30]. Therefore, the process of dealing with complex emotions in the psychological intervention of these callers should not be ignored. Just as Rubin et al. suggested, governments urgently need guidance and actionable information on effective public health and psychological interventions to protect the public’s mental health [31].

Moving on to the reasons for calling, the most frequently mentioned problem is emotional distress, followed by the need for practical help and sleep problems. Among emotional issues, the most important reason for urging them to call is that the epidemic made them worry about themselves and their family members. They did not know how to control themselves from not concerning about the epidemic. Someone even told us on the hotline that they felt desperate when they saw the newly increased number of infected patients. We found that most callers called us to ask for specific information about the epidemic. They wanted to know when the epidemic would end and what they could do to protect themselves from infecting. Additionally, many callers also wanted to know the working time of our hospital so that they could get some sleeping pills or take psychotherapies. When asked about the reasons, most of them believed that the epidemic had negative effects on them, and they wanted to relieve themselves or evaluate whether they had depression. Moreover, many callers also reported sleep problems. The most common reason is that the epidemic disrupted their routines, and anxiety about the epidemic made it difficult for them to sleep. We can see that due to the epidemic, the way of getting medical treatment and psychotherapy has changed a lot. Many people preferred to choose the hotline to seek mental health and medical-related services. According to Corruble’s report, the pandemic has required a shift of 90% of their outpatient activity to telepsychiatry, and he suggested that a dedicated hotline for psychiatry teleconsultation was necessary [32]. Through our investigation, we call on the government to establish a hierarchical psychological assistance system. As the telephone is a familiar and reliable technology, it is adequate for many COVID-19 related conversations. Patients who would like to obtain general information about coping with stress during the epidemic should be redirected to a similar hotline. Those with milder and more complicated symptoms during the epidemic can be transferred to the video call conducted by professional psychotherapists [33].

Finally, through the hotline service feedback, the study found that the hotline can relieve the emotions of many people to a certain extent and effectively solve some medical-related problems. Our result also discovered that as the pandemic progressed, the public reported the persistent effectiveness of the hotline, which indicated that the hotline could be a powerful way to provide help during the pandemic. It helped the government further reduce the proportion of patients suffering from severe mental illness due to epidemics and provided some ideas on using modern technologies that effectively provide treatment during epidemics. Perhaps in the near future, we could use a combination of hotline and computer video calls and even provide medical services to the public through mobile applications [34].

### Limitation

Although this study provides some valuable information on the mental status of the public during the COVID-19 epidemic, its research data is relatively subjective and lacks objective scores to assess their mental health accurately. In addition, the effectiveness of the hotline was evaluated through verbal feedback from the callers. However, due to the limitations of the hotline, this study could only obtain such data. In the future, we look forward to obtaining more detailed objective data from other sources (such as the Internet), which may help us analyze the psychological state of the public at the early stage.

## Conclusion

Through this research, we found that during the epidemic, the emotions that made the public call the hotline were mainly manifested as anxiety and fear, and more than half the callers attempted to seek support around their emotion. The most concern is still related to epidemics and its adverse effects on the public. Fortunately, facts have proved that the psychological assistance hotline is an active and effective rescue measure in this case.

## Data Availability

The datasets used and/or analysed during the current study are available from the corresponding author on reasonable request.
